# Conventional and Microwave Hydrothermal Synthesis and Application of Functional Materials: A Review

**DOI:** 10.3390/ma12071177

**Published:** 2019-04-11

**Authors:** Guijun Yang, Soo-Jin Park

**Affiliations:** Department of Chemistry, Inha University, 100 Inharo, Incheon 402-751, Korea; yanggj91@gmail.com

**Keywords:** hydrothermal method, microwave hydrothermal method, functional materials, application

## Abstract

With the continuous development and progress of materials science, increasingly more attention has been paid to the new technology of powder synthesis and material preparation. The hydrothermal method is a promising liquid phase preparation technology that has developed rapidly during recent years. It is widely used in many fields, such as the piezoelectric, ferroelectric, ceramic powder, and oxide film fields. The hydrothermal method has resulted in many new methods during the long-term research process, such as adding other force fields to the hydrothermal condition reaction system. These force fields mainly include direct current, electric, magnetic (autoclaves composed of non-ferroelectric materials), and microwave fields. Among them, the microwave hydrothermal method, as an extension of the hydrothermal reaction, cleverly uses the microwave temperature to compensate for the lack of temperature in the hydrothermal method, allowing better practical application. This paper reviews the development of the hydrothermal and microwave hydrothermal methods, introduces their reaction mechanisms, and focuses on the practical application of the two methods.

## 1. Introduction

**Figure 1:**
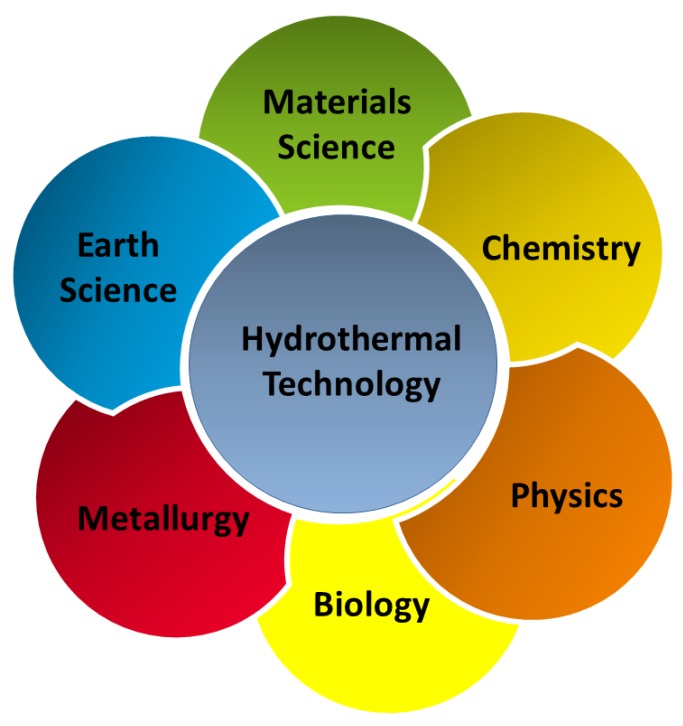
Application field of hydrothermal methods.

During the process of continuous development of materials science, the research and development of new processes for material preparation and synthesis has always been an important part. For a long time, researchers have been searching for a material synthesis method with limited pollution, easy operation, excellent product performance, and low production cost [1,2,3]. The synthesis methods of inorganic powder materials mainly include the solid phase, liquid phase, and gas phase methods [4,5]. The solid phase method results in a high yield and is easy to realize for large-scale industrial production. However, because of the limitations of the equipment and the process itself, it is difficult to control the particle size, purity, and morphology of powder using the solid phase method [6,7,8,9,10]. The liquid phase method mainly includes the precipitation [11,12,13], hydrothermal [14,15,16], colloidal [17,18,19], and sol-gel methods [20,21,22]. The advantages of the liquid phase method are convenient operation, simple synthesis process, and controllable particle size. However, most liquid phase methods consume more energy and have high costs. The gas phase process generally includes the evaporation-condensation [23,24,25] and chemical vapor phase reaction methods [26,27,28]. The particles prepared using the gas phase method are small in size, have a high uniform purity, and have high surface activity and good dispersibility, but equipment and a large amount of solvent is required during the reaction process, making it difficult to produce at a large scale. The hydrothermal method is a preparation method that has been further researched during recent years. The hydrothermal method is a type of soft chemical synthesis method developed by simulating the formation process of some ores in nature. It can be used to grow a variety of single crystals to prepare ultrafine agglomerated or less agglomerated crystallized ceramic powders to complete certain organic reactions, treat some organic waste materials that endanger the human living environment, or sinter some ceramic materials at a relatively low temperature. Nowadays, hydrothermal technology has found its place in several branches of science and technology, covering a range of fields, such as materials science, earth science, metallurgy, physics, chemistry, biology, etc. (Figure 1). Given the wide application and increasing importance of the hydrothermal method, it has been considerably improved during a process of continuous development. For example, the use of microwaves [29,30], mechanical mixing [31,32], and electric fields [33,34] to enhance the reaction kinetics of the hydrothermal method has resulted in widespread attention. With these techniques, the experimental time has been reduced by at least two orders of magnitude, making this technology more economical and practical. Among them, the microwave hydrothermal method has been a high-profile research topic during recent years. As a development of the hydrothermal method, it is more widely used in ceramics preparation. The microwave temperature successfully compensates for the temperature unevenness during the hydrothermal process. In addition, the crystal size, morphology, and agglomeration of ceramic oxides can be controlled by adjusting the ratio of starting materials, pH of the reaction system, time, and temperature of the reaction. This paper reviews the development of the hydrothermal and microwave hydrothermal methods, introduces their reaction mechanisms, and emphatically introduces the application of the two methods to advanced functional materials.

## 2. Hydrothermal Method

**Figure 2:**
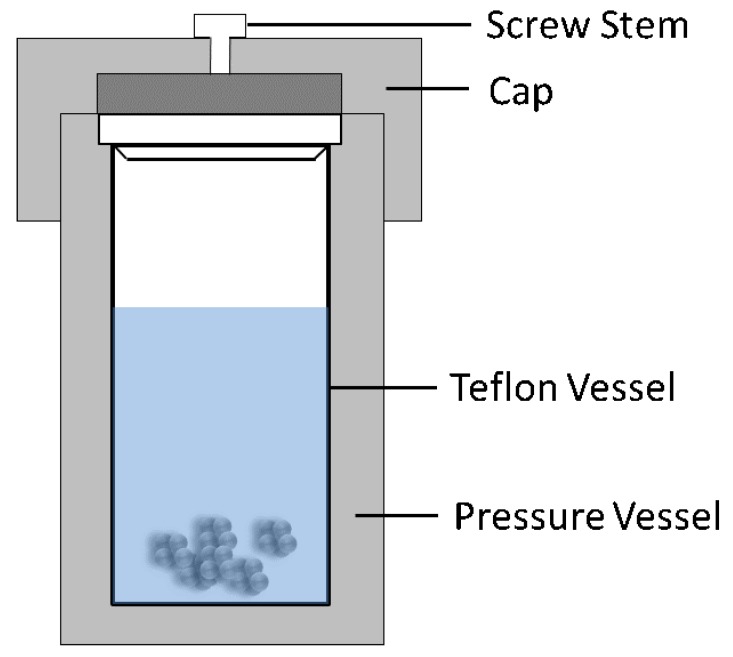
Schematic of typical hydrothermal method equipment.

**Figure 3:**
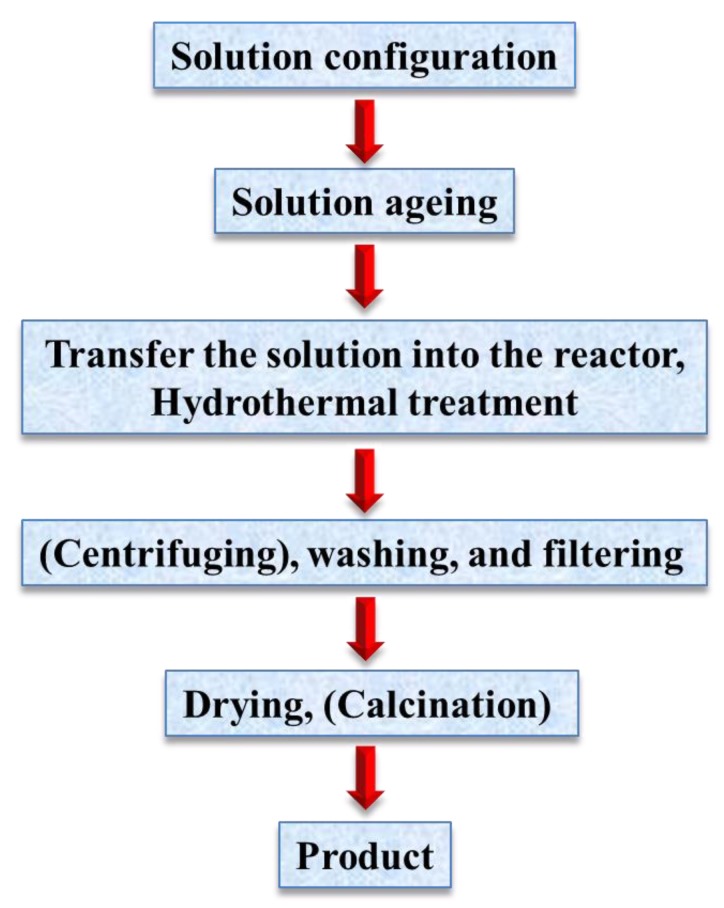
General steps for hydrothermal preparation.

The term, hydrothermal, originated from geology, beginning during the mid-19th century when geologists simulated hydrothermal conditions to study the formation of certain minerals and rocks. On this basis, hydrothermal methods began to be applied to single crystal growth; powder preparation has been developed for nearly 200 years. The hydrothermal method refers to the use of an aqueous solution as a reaction system in a special closed reaction vessel (Figure 2) to create a high-temperature, high-pressure reaction environment by heating the reaction system and pressurizing it (or the vapor pressure generated by itself). The process dissolves and recrystallizes a substance that is poorly soluble or insoluble under normal conditions [35,36]. The general preparation steps of the hydrothermal method are shown in Figure 3.

### 2.1. Reaction Kinetics and the Crystal Growth Mechanism of the Hydrothermal Method

The main steps of crystalline growth under hydrothermal conditions are as follows: First, the reactants are dissolved in a hydrothermal medium and enter the solution in the form of ions or molecular groups. Second, the ions or molecules are separated by the temperature difference between the upper and lower portions of the kettle. The ions or molecular groups are transported to the low-temperature region, where the seed crystal is grown to form a supersaturated solution. Third, the ions or molecular groups are adsorbed, decomposed, and desorbed at the growth interface. Fourth, the adsorbed material moves at the interface. Finally, the dissolved matter crystallizes. The crystal morphology of crystals under hydrothermal conditions is closely related to the growth conditions [37,38,39]. The same crystals may show different morphologies under different hydrothermal conditions. It is very important to study the crystal morphology to predict the crystal growth mechanism. The theoretical model of crystal morphology mainly includes the Bravais-Friedel-Donnay-Harker (BFDH) rule [40] and periodic bond chain (PBC) theory [41,42]. The BFDH rule starts from the density of the surface network of the crystal and considers the influence of the screw dislocation and slip on crystal structure morphology, providing the ideal growth morphology of the crystal. The PBC theory quantitatively describes the crystal growth morphology from the perspective of intermolecular bond properties and binding energies. However, both theoretical models do not consider the influence of changes in physical and chemical conditions (such as the temperature, pressure, and solvent) on the crystal morphology during crystal growth and cannot explain the growth characteristics of polar crystals. Therefore, a theoretical model of “growth primitives” was developed based on a large number of experiments [43]. The “growth primitive” theory holds that in the transport phase of the second step, the ions or molecular groups dissolved in solution react to form a polymer with a certain geometric configuration for a long period of time. The size and structure of the long primitive element are related to the hydrothermal reaction conditions. In a hydrothermal reaction system, there are many forms of growth primitives and a dynamic balance is established among them. The more stable a growth cell is, the more likely it is to appear in the system. From the viewpoint of crystallography, the positive ions of the growth element are associated with negative ions that satisfy certain coordination requirements, and thus are further referred to as “negative ion coordination polyhedral growth elements”.

### 2.2. The Role of Water in the Hydrothermal Method 

In a hydrothermal reaction, water can participate in the reaction as a chemical component or it can be a solvent or a puffing accelerator [44,45,46]. As a pressure transmission medium, the formation of inorganic compounds can be achieved by accelerating the osmotic reaction and controlling the physical and chemical factors of the process. In high-temperature and high-pressure hydrothermal systems, the properties of water will produce the following changes: (1) The ionic product increases and the ionic product of water rapidly increases with the increase in pressure and temperature. Under high-temperature and high-pressure hydrothermal conditions, the hydrolysis reaction and ion reaction rates will naturally increase with water as the medium. According to the Arrhenius equation, dlnk/dt = E/RT^2^, the reaction rate constant has an exponential function with increasing temperature. Therefore, the main reason for the increase in the hydrothermal reaction is that the ionization constant of water increases as the reaction temperature and pressure increase. (2) The viscosity and surface tension of water decrease as the temperature increases. In hydrothermal systems, the viscosity of water decreases and the mobility of molecules and ions in solution greatly increases, such that crystals grow under hydrothermal conditions more rapidly than under other conditions. (3) The dielectric is often low and the dielectric constant generally decreases with increasing temperature and increases with increasing pressure. Under hydrothermal conditions, the reaction is mainly affected by temperature and the dielectric constant of water is significantly reduced. This decrease affects the ability and behavior of water acting as a solvent. (4) The density decreases, and properties, such as the viscosity, dielectric constant, and solubility of the material, increase with increasing density while the diffusion coefficient decreases with increasing density. (5) The vapor pressure increases and accelerates the reaction by increasing the chance of collision among molecules. 

### 2.3. Role of Mineralizer

Because of the low solubility of the compounds involved in the hydrothermal method in water, even if the hydrothermal reaction temperature is very high, the solubility of most substances in pure water will not exceed 0.1 to 0.2 wt.%. Therefore, one or several substances are often introduced into the system to increase the solubility during the crystal growth process. These substances are termed “mineralizers”. Mineralizers are generally a class of compounds whose solubility in water continues to increase with increasing temperature, such as some low melting salts, acids, and bases [47,48,49,50]. The addition of a suitable mineralizer not only increases the solubility of the solute in the hydrothermal solution, but also changes its solubility temperature coefficient. Some mineralizers can also form complexes with the crystalline material to accelerate the crystal nucleation rate. In addition, the type of mineralization agent also has a great influence on the quality and growth rate of the crystal. Studies have shown that the use of acids, such as HCl, H_2_SO_4_, H_3_PO_4_, HNO_3_, and HCOOH, as mineralizers can reduce the crystal growth temperature to below 300 °C, thus allowing the use of relatively simpler autoclaves [51,52]. Tani et al. [53] summarized the effect of mineralizers on ZrO_2_ crystallization using the hydrothermal method as shown in Table 1. The as-prepared ZrO_2_ exhibits a different morphology and crystalline size when using different mineralizers.

### 2.4. Basic Classification of Hydrothermal Reactions

According to the research object and purpose, the hydrothermal method can be divided into hydrothermal crystal growth, synthesis, reaction, and treatment, which are used to grow various singular crystals, prepare functional ceramic powders, and complete some organic reactions or treatment of some organic wastes that endanger the environment, as well as the sintering of certain ceramic materials at relatively low temperatures. According to the reaction temperature, it can be divided into a low-temperature hydrothermal method and supercritical hydrothermal method. According to the equipment differences, it can be divided into the ordinary hydrothermal method and a special hydrothermal method. The so-called special hydrothermal method refers to the addition of other action fields to the hydrothermal reaction condition system, such as direct current electric, magnetic, and microwave fields.

## 3. Microwave Hydrothermal Method

Microwave performance studies began during the 1930s. Initially, microwave applications remained in the radio field. During the 1960s, researchers discovered the thermodynamic effects of microwaves and developed a new field of research. In the early 1990s, Dr. Komarneni of the University of Pennsylvania became a pioneer by comparing the differences between traditional hydrothermal synthesis and special hydrothermal (microwave hydrothermal) synthesis [54]. Nowadays, the microwave thermal effect has been applied in various fields and its development prospect is very broad. 

### 3.1. Reaction Mechanism of Microwave Heating

**Figure 4:**
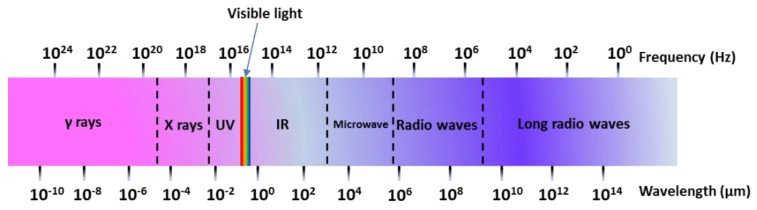
Spectral wavelength-frequency diagram.

**Figure 5:**
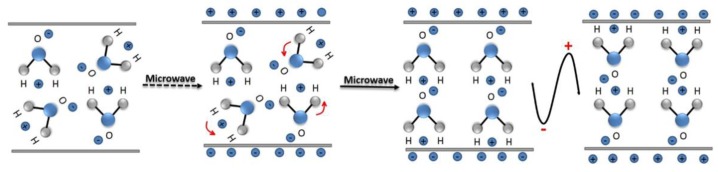
Schematic diagram of water molecular motion in a microwave field.

A microwave (MW) is a form of electromagnetic energy associated with electromagnetic fields. Microwave refers to an electromagnetic wave with a wavelength between 300 MHz and 300 GHz in the range of 1 m to 1 mm (Figure 4). When it is irradiated on the surface of the medium, a small part of it will be reflected, and most of it can penetrate into the interior of the medium and be gradually absorbed by the medium and converted into heat energy. Between infrared and radio waves, only microwaves with frequencies concentrated at 900 MHz and 2.45 GHz can be used for heating. According to the interaction of microwaves with the material, materials can be characterized as three types: Transparent (low dielectric loss material), with little attenuation (if any) when microwaves pass, such as non-metallic materials and most polymer materials; opaque (conductor), microwave reflection without penetration, such as metals and alloys; absorption (high dielectric loss material), it is based on the value of the dielectric loss factor, which absorbs microwave energy to some extent, such as water. There are many mechanisms for the interaction between microwaves and matter, which can be summarized as dielectric loss, conduction loss, and magnetic loss. These mechanisms apply to some common forms of heating, such as medium heating, Joule heating, and induction heating, all of which rely on electromagnetic field characteristics and material properties [55,56]. Electromagnetic fields can be divided into electric and magnetic fields, and fields with different properties interact with materials according to different mechanisms. The electric field of the microwave is responsible for the heating of the medium. In the microwave frequency range, medium heating is mainly carried out by two mechanisms of dipole polarization and ion conduction [57]. For example, microwaves can be used in the field of chemical synthesis because the reactants involved in chemical reactions carry various polar molecules, such as water, alcohols, and carboxylic acids. Under normal circumstances, these molecules are in a disorderly state of motion. When the microwave oven magnetron radiates a very high frequency microwave, the microwave energy field continuously changes hundreds of millions of times per second, and the positive and negative polarities are at the same frequency. In exchange, the molecular motion undergoes tremendous changes; from an original chaotic motion trajectory to an ordered high-frequency vibration, resulting in collision, friction, and extrusion, such that kinetic energy-microwave energy is converted into heat energy. The molecular heating principle of polar molecules is shown in Figure 5. Moreover, magnetic field heating also affects microwave heating, and the literature shows that for some magnetic medium materials (ferrite) [58,59], the effect of the microwave magnetic field is stronger than the one from the electric field. Furthermore, some literature has reported non-thermal phenomena that are widely known as “microwave effects” that also affect microwave heating, such as an increase of the reaction rate of the thermosetting resin by microwave curing and an increase of the densification speed of ceramic sintering [60].

### 3.2. Characteristics of the Microwave Hydrothermal Method

The microwave hydrothermal method is a new type of method developed during recent years for preparing powder. It uses microwaves for the heating method using the principle of the hydrothermal method, but it is different from the traditional hydrothermal synthesis method. The microwave hydrothermal method is a combination of the hydrothermal and microwave methods, fully exerting the advantages of microwaves and water heat. Compared to the hydrothermal method, the microwave hydrothermal heating method is no longer a single conduction method, but is heated by microwaves. Even if the sample has a certain depth, it can be penetrated by microwaves and each depth can be heated at the same time, avoiding heat conduction, resulting in a temperature difference, and greatly improving the reaction speed. Compared with the traditional hydrothermal method, the microwave hydrothermal method has the characteristics of a fast heating speed, sensitive reaction, and uniform heating system, so that it can rapidly prepare nanoparticles with a narrow particle size distribution and uniform morphology. Therefore, for the reaction with a high reaction temperature difference and long reaction time, the microwave hydrothermal method can be used to prepare samples that take a long time or are sensitive to the temperature difference. In addition, the microwave hydrothermal method has great potential research and application value for the preparation of ultrafine powder. Microwave-assisted synthesis is generally faster, cleaner, and more economical than traditional hydrothermal methods. 

## 4. Application

Hydrothermal methods can transform a metal alloy into an ultrafine powder under certain conditions and also react a solution containing various metal ions to form a crystalline powder under high temperatures and high pressures. With the advancement in technology, it is widely used in various fields.

### 4.1. Simple Oxides

Powder synthesized using the hydrothermal method has the characteristics of fine crystals, controllable morphology, high purity, and a narrow grain size distribution. It has been widely used in the preparation of various powders during recent years. Simple oxides, such as ZrO_2_ [53,61], Al_2_O_3_ [62,63], Fe_2_O_3_ [64,65], MnO_2_ [66,67], MoO_3_ [68,69], TiO_2_ [70,71], ZnO [72,73], CuO [74,75], CeO_2_ [76,77], Nb_2_O_5_ [78,79], etc., have been prepared using the hydrothermal method and reported in detail. Roy et al. [80] obtained TiO_2_ with five different morphologies (nanorods, nanocapsules, nanoellipsoids, nanosheets, and nanocuboids) using the hydrothermal method through the adjusted molar concentration ratio of TBAH (tetrabutyl ammonium hydroxide) to DEA (diethanolamine). The solution of TBAH and TTIP (titanium tetraisopropoxide) in the stoichiometric ratio with a different content of DEA was transferred to a Teflon-lined stainless-steel autoclave, reacted at 200 °C for 5 h, and naturally cooled to room temperature. After several rinses, the product was dried at 60 °C for 24 h under an air atmosphere. Zavala et al. [81] prepared TiO_2_ nanotubes using dispersed commercial Degussa P25 TiO_2_ nanoparticles in a NaOH solution and then reacted it in an autoclave at 110 °C for 72 h. The obtained precursor was dispersed in diluted HCl solution and the suspension was centrifuged and washed to obtain the final product with a nanotube morphology. As a development of the hydrothermal method, microwave synthesis technology can synthesize high purity and fine particle size powders in a short time and avoid the agglomeration often caused by traditional heating. Komarmeni et al. [82] first reported the synthesis of crystalline unit oxides, such as TiO_2_, ZrO_2_, and Fe_2_O_3_, as well as binary oxides, such as KNbO_3_ and BaTiO_3_, using the microwave hydrothermal method. The effects of different parameters, such as chemical concentration, time, and temperature, on crystallization kinetics under microwave-hydrothermal conditions at a microwave frequency of 2.45 GHz were studied. Wilson et al. [83] compared titanium dioxide prepared using an ordinary hydrothermal method to that using a microwave hydrothermal method. The results show that the crystallinity of the microwave-treated TiO_2_ colloid is obviously higher than that of the ordinary hydrothermal treated TiO_2_. However, the time required and the energy consumed are significantly less than that of the hydrothermal convection treatment. Yap et al. [84] reported a comparative study of the hydrothermal and microwave hydrothermal methods by synthesizing CuO crystal on a cellulose matrix. Compared to the hydrothermal method, the CuO crystal obtained using the microwave hydrothermal method transformed into a Cu_2_O crystal and the obtained Cu_2_O crystal showed different morphologies and sizes. We selected several materials to compare the morphology, particle size, and reaction conditions using hydrothermal and microwave hydrothermal methods (Table 2) [62,63,85,86,87,88,89,90,91,92,93,94,95]. According to the results, the traditional hydrothermal method typically requires longer reaction times, higher energy consumption, or special conditions compared to the microwave hydrothermal method. Therefore, the microwave hydrothermal method shows a relatively strong advantage when preparing some materials with special requirements.

### 4.2. Mixed Oxides—Perovskite

Perovskites are the most abundant minerals on the planet and were originally referred to as CaTiO_3_. The structural formula of the perovskite oxide material is ABO_3_, where A is a rare earth metal or an alkaline earth metal and B is a first row transition metal [96]. Perovskite oxide crystals are anisotropic because of their deviation from the ideal crystalline structure, causing ferroelectricity, piezoelectricity, and giant magnetoresistance, which can be widely used in information storage and piezoelectric sensors. Hydrothermal synthesis is an effective means to prepare multi-component oxide ceramic powders, including perovskite. However, many water reactions are heterogeneous and the reactions between solid and liquid phases or between liquid and liquid phases determine the properties of materials. Therefore, by optimizing reaction conditions, such as temperature and reactant concentration, the property of a powder can be controlled. Han et al. [97] compared the photocatalytic activity of CaTiO_3_ synthesized using solid state, sol-gel, and hydrothermal methods. The results showed that CaTiO_3_ prepared using the hydrothermal method had the highest photocatalytic activity of the three samples, lower than that of TiO_2_. Gonçalves et al. [98] prepared Pr-doped CaTiO_3_ using the polymeric precursor and microwave-assisted hydrothermal methods. Chybczynska et al. [99] obtained BiFeO_3_ powder with a spherical flower structure by the microwave hydrothermal method. The special flower microcrystalline structure, for example, the grain size and shape, exhibited a positive effect on the dielectric response and electrical conductivity of BiFeO_3_ ceramics. Liu et al. [100] synthesized BaTiO_3_ from (BaNO_3_)_2_, TiCl_4_, and KOH using the conventional hydrothermal method (CH) and microwave-hydrothermal (MH) method. Additionally, the sinterability, microstructure, and dielectric properties of the samples prepared by the two methods were compared. The results show that the sintering properties and dielectric properties of BaTiO_3_ prepared by the MH method and the CH method are basically the same. However, compared with the CH method, the MH method greatly shortens the time and saves energy. It can be seen from the experimental comparison that microwave irradiation has a significant effect on the morphology of a material because the high-energy microwaves lead to rapid heat conduction. In addition, microwave irradiation has a short heat treatment time and low temperature, making it possible for use in large-scale industrial production application.

### 4.3. Bioceramics

Bioceramics are a new class of ceramic materials related to organisms or biochemistry. They can be directly used in the human body or related medical or biological materials. As a biomedical material, ceramics are required to be of high purity and have good biocompatibility. They can be used to create hard tissue substitution materials, such as artificial bones, joints, and teeth. According to the activity in vivo, bioceramics can be divided into three types: Bioabsorbent, bioactive, and bioinert ceramics. At present, the most commonly used absorbable bioceramics are mainly represented by β-tricalcium phosphate (β-TCP) and calcium sulfate bioceramics; the most representative bioactive ceramics include bioactive glass and hydroxyapatite ceramics. Since the rise of ceramic powder prepared using the hydrothermal method during the 1970s, it has been highly valued by many countries in the world, particularly industrialized countries. Suchanek et al. [101] developed a hydroxyapatite coating consisting of microcrystalline hexagonal crystals grown on a Ti/TiO_2_ matrix using the hydrothermal method. Two different reagents were used in the hydrothermal process: Ethylenediaminetetraacetic acid and monoethanolamine. The results show that the monoethanolamine-assisted hydrothermal method showed better performance than that of the others. Usually, hydroxyapatite crystals are needle-like crystals. If the crystal morphology of hydroxyapatite can be controlled in a tube, flaky, or other three-dimensional structure, this will expand its application. Kamitakahara et al. [102] successfully prepared tubular hydroxyapatite using a hydrothermal reaction with calcium phosphate and anatase as reactants. During the hydrothermal process, anatase particles controlled the nucleation and crystal growth of the hydroxyapatite, which was beneficial to the formation of tubular hydroxyapatite. Jokic’a et al. [103] synthesized hydroxyapatite whiskers using Ca(NO_3_)_2_·4H_2_O, (NH_4_)_2_HPO_4_·2H_2_O, and urea as reactants. The morphology of the hydroxyapatite can be controlled by the initial concentration of the solution and the amount of urea. The obtained hydroxyapatite particles have a sheet morphology formed by the center of a hexagonal cup shape. The refinement of the structure of the hydroxyapatite whisker determines that the hexagonal crystalline structure has a strong preferred orientation along the c-axis direction, which is significantly different from that of the commonly observed random crystal orientation. There are many means to synthesize hydroxyapatite, but only a few methods can control the product morphology. Unlike traditional hydrothermal methods, Yu et al. [104] prepared ultralong hydroxyapatite nanowires using sodium oleate, CaCl_2_, and NaH_2_PO_4_·2H_2_O as starting materials using the microwave-assisted hydrothermal method. This reaction can be completed over a short period of time (within 20 min); compared to the traditional hydrothermal method, the synthesis time is greatly reduced by approximately two orders of magnitude, improving the efficiency and conserving energy. Kumar et al. [105] reported the rapid synthesis of mesoporous hydroxyapatite nanocrystals with a controlled size, morphology (needle-like, rod-like, and fiber-like), and surface area using the microwave hydrothermal method. 

### 4.4. Thin Films

Hydrothermal preparation of thin films is a promising liquid phase membrane-forming technique developed during recent years. The chemical reaction of hydrothermal preparation of thin films generally uses inorganic salts or aqueous hydroxide solutions as precursors, using single crystal silicon, metal flakes, α-Al_2_O_3_, glass slides, and plastics as substrates at low temperatures (often below 300 °C). The substrate solution impregnated with the substrate is subjected to an appropriate hydrothermal treatment to form a stable crystalline phase film on the substrate. Hydrothermal preparation of the film does not require post-crystallization heat treatment, avoiding defects, such as curling, cracking, grain coarsening, or film reaction with the substrate or atmosphere during the heat treatment process. Moreover, the hydrothermal method for preparing the film is simple; the hydrothermal treatment temperature is low and mutual diffusion of the film and the substrate component before and after the hydrothermal treatment is avoided. The obtained film is of high purity and has good uniformity. There are many types of films that can be synthesized using the hydrothermal and microwave hydrothermal methods, such as BaTiO_3_ [106,107], SrTiO_3_ [108,109], ZnO [110,111], TiO_2_ [112,113], etc. Wu et al. [114] reported a simplified hydrothermal method to grow high-quality ZnO thin films on (0001) sapphire substrates. The prepared ZnO thin films showed a large area, smooth surface, and no obvious fluctuation, and are available for fabricating application devices. Zhou et al. [115] grew a BaTiO_3_ film on fluorine-doped tin oxide-coated patterned silicon wafers using a two-step hydrothermal method. The prepared thin films showed a larger surface area than that of conventionally deposited piezoelectric films, obtaining a higher storage density of electronic devices. Zhang et al. [116] synthesized TiO_2_ thin films via hydrolysis of titanium butoxide and the peptization process with no hydrothermal process, convection hydrothermal method, and microwave hydrothermal method. The results showed that the microwave hydrothermal treatment is expected to replace the traditional hydrothermal treatment, providing electrodes with higher photocatalytic properties for the oxidation process of adsorption-based organic compounds.

### 4.5. Vanadates

Vanadate is a general term for pentavalent vanadium oxyacid salts, mainly including orthovanadate (MVO_4_), pyrovanadate (MV_2_O_7_), and metavanadate (MVO_3_). These salts can be prepared from a solution; for example, V_2_O_5_ is dissolved in a concentrated NaOH solution to obtain a colorless Na_3_VO_4_ solution in which vanadium is present in the form of orthovanadate VO4^3−^. In an aqueous solution, as the acidity of the solution increases, the vanadate will undergo different degrees of condensation to form different composition polyanions. Metal vanadate is widely used. For example, it can be used as a good matrix material in the field of fluorescent and laser materials. It can also be used as an electrode material for batteries. It also plays an important role in the field of catalysis. Prado-Gonjal et al. [117] used industrial vanadium powder as a raw material to prepare H_2_V_3_O_8_ nanobelts for electrode materials by the microwave hydrothermal method. Compared with the conventional hydrothermal method reported in the past, the proposed method is faster, cheaper, and more environmentally friendly, and is suitable for large-scale application as an electrode material. Xu et al. [118] constructed a carbon nanotube (CNT)/LaVO_4_ nanostructure for efficient antibiotic photodegradation. The LaVO_4_ coating layer prepared using the hydrothermal method was uniformly coated on the surface of the CNT. Li et al. [119] reported a novel flower-like BiVO_4_ and BiVO_4_/Bi_2_Ti_2_O_7_ heterojunction photocatalyst using a surfactant-free hydrothermal method. A mixed aqueous solution of Bi(NO_3_)_3_·5H_2_O and Na_3_VO_4_·12H_2_O was transferred to a sealed Teflon autoclave and heated at 160 °C for 12 h. Similarly, Lin et al. [120] also prepared monoclinic BiVO_4_ using the hydrothermal method without surfactant. The experiment used Bi(NO_3_)_3_·5H_2_O, NH_4_VO_3_, and nitric acid as raw materials and adjusted the pH with ammonia water. The obtained solution was placed in a Teflon autoclave at 180 °C for 24 h. Liu et al. successfully synthesized sandwich-shaped BiVO_4_ flakes using Bi(NO_3_)_3_·5H_2_O, NH_4_VO_3_, and polyethylene glycol with a molecular weight of 10,000 (PEG-10000) as raw materials using the microwave hydrothermal method. The mixed solution of the raw materials was transferred to a Teflon autoclave and then heated in a microwave at 140 °C for 4 h. Finally, the resulting powder was calcined at 450 °C to obtain sandwich-like BiVO_4_ sheets. Kshetri et al. [121] synthesized Yb^3+^- and Er^3+^-doped YVO_4_ nanoparticles with efficient near-infrared to visible upconversion properties using the microwave hydrothermal method. The experiment was conducted for 1 h at a low temperature of 140 °C and then calcination was conducted at 300 °C for 4 h to obtain the target product. YVO_4_ nanoparticles showed good crystallinity and a uniform particle size (~100 nm). 

### 4.6. Garnets

The hydrothermal method as a method to grow singular crystals from aqueous solution in a closed high-pressure vessel simulates the natural process of hydrothermal mineralization and crystallization to a certain extent. Laudise et al. [122] used Y and Fe oxides as raw materials to study the growth and crystallization of yttrium iron garnet (Y_3_Fe_5_O_12_, YIG) crystals under hydrothermal conditions. They believe that YIG is stable only in an extremely narrow temperature range of approximately 725 °C. The results show that the alkali (NaOH) concentration and temperature have a significant effect on the product phase development. Cho et al. [123] synthesized cubic YIG using nitrate and NH_4_OH as raw materials under low-temperature hydrothermal conditions of 225 °C and studied the effect of process parameters, such as mineralizer, temperature, and nonstoichiometry, on the reaction products and morphology. The results showed that the YIG phase tends to disappear when the temperature increases to 250 °C. Moreover, if an excessive amount of Y is added, the yield of the YIG phase can be effectively increased. The isometric YIG particles at 225 °C are transformed into irregular star particles. The YIG phase was found to be unstable in NaOH. Ramesh et al. [124] synthesized gadolinium iron garnets (GdIGs) using the microwave hydrothermal method at 160 °C for 45 min. The obtained powder has high crystallinity and no obvious agglomeration and shows high reactivity. Sadhana et al. [125] prepared Dy^3+^ doped Y_3−x_Dy_x_ Fe_5_O_12_ (x = 0–3) nanopowders using the microwave hydrothermal method. The microwave treatment was conducted at 200 °C for 45 min with a power of 600 W. Compared to the common hydrothermal method, the microwave temperature method can significantly decrease the reaction temperature and time.

## 5. Conclusions

The hydrothermal method has many advantages; thus, it has shown great potential in the preparation of ceramic oxides, bioceramics, thin films, vanadates, garnets, and others. In general, hydrothermal preparation materials remain in the active exploration and developmental stage. There are few hydrothermal studies regarding non-aqueous solvent systems. Most of the materials prepared using the hydrothermal method are oxides and oxygenated salts. Therefore, research regarding the physicochemical properties of hydrothermal devices, solvents, and mineralizers, as well as the study of the mechanisms of chemical reactions during hydrothermal processes, are of great significance in promoting the preparation of materials using hydrothermal methods. With the development of modern materials science and engineering research, the application field and basic theory of hydrothermal methods will be further developed. Simultaneously, with the intersection of disciplines, the combination of the hydrothermal method with other methods will be a developmental trend, which will result in the hydrothermal method being more widely used.

## Figures and Tables

**Table 1:** Effect of mineralizers on ZrO_2_ crystallization using the hydrothermal method.

Mineralizer	ZrO_2_ (300 °C, 24 h, 100 Mpa)	
	Tetragonal	Monoclinic
KF (8 wt.%)	No data	16 nm
NaOH (30 wt.%)	No data	40 nm
H_2_O	15 nm	17 nm
LiCl (15 wt.%)	15 nm	19 nm
KBr (15 wt.%)	13 nm	15 nm

**Table 2:** Comparison of the morphology, particle size, and reaction conditions using the hydrothermal and microwave hydrothermal methods.

	Hydrothermal Method					Microwave Hydrothermal Method				
	Morphology	Raw Materials	Conditions	Size	Ref.	Morphology	Raw Materials	Conditions	Size	Ref.
ZrO_2_	Spherical	ZrOCl_2_·8H_2_O, NH_4_OH, NaOH	150 °C, 24 h	20–30 nm	[87]	Monoclinic	ZrOCl_2_·8H_2_O, NaOH	200 °C, 2 h, 2.45 GHz	10–20 nm	[88]
	Rod	ZrOCl_2_·8H_2_O, NH_4_OH, NaOH	200 °C, 24 h 250 °C, 24 h	50 nm × (200–400) nm 80 nm × (200–500) nm	[87]	Tetragonal-monoclinic	ZrOCl_4_, NaOH	150–220 °C, 30 min	~20 nm	[89]
Al_2_O_3_	Hollow	Al(NO_3_)_3_·9H_2_O, glucose	160 °C, 3–8 h	5.4–6.9 μm	[64]	Hollow	KAl(SO_4_)_2_·12H_2_O, CO(NH_2_)_2_	180 °C, 40 min, 300 W	0.8–1.2 μm	[90]
	Rod	Al(NO_3_)_3_·9H_2_O, N_2_H_4_⋅H_2_O	200 °C, 12 h	8 nm × (220–532) nm	[65]	Fiber	Surfactant Brij 56, H_2_SO_4_, Aluminum sec-butoxide	80 °C, 30 min, 500 W	~50 nm	[91]
MnO_2_	Belt	Mn_2_O_3_, NaOH	170 °C, 12 h	5–15 nm	[92]	Flower Nanosheet Fiber	KMnO_4_, HCl	100 °C, 25 min 140 °C, 25 min 180 °C, 25 min	200–400 nm 10 nm 2–6 μm	[94]
	Urchin Urchin Nanowire	MnSO_4_, (NH_4_)_2_S_2_O_8_	80 °C, 4 h 110 °C, 4 h 140 °C, 4 h	2–3 μm 30–40 μm ultrathin	[83]	Nanosphere	KMnO_4_, MnSO_4_·H_2_O	75 °C, 30 min	70–90 nm	[95]
TiO_2_	Nanotube	TiO_2_, NaOH	150 °C, 48 h	8.1–27.3 nm	[96]	Nanowire	TiO_2_, NaOH	210 °C, 2 h, 350 W	80–150 nm	[96]
	Acicular	TiOCl_2_	195 °C, >8 h	100 nm × 50 nm	[97]	Spherical	TiOCl_2_	195 °C, >30 min, 2.45 GHz	10 nm	[97]
